# Schizophrenia moderates the relationship between white matter integrity and cognition

**DOI:** 10.1016/j.schres.2018.03.033

**Published:** 2018-09

**Authors:** Luis F.S. Castro-de-Araujo, Mathew Allin, Marco M. Picchioni, Colm Mcdonald, Christos Pantelis, Richard A.A. Kanaan

**Affiliations:** a CAPES Foundation, Ministry of Education of Brazil, Brasília-DF, Brazil; b University of Melbourne, Department of Psychiatry, Austin Health, Heidelberg, Victoria, Australia; c Institute of Psychiatry, King's College, London, UK; d Department of Psychosis Studies, Institute of Psychiatry, Psychology & Neuroscience, King's College London, London, UK; e National University of Ireland (NUI), Galway, Ireland; f Department of Psychiatry, University of Melbourne, Parkville, VIC, Australia

**Keywords:** Cognitive tests, Linear models, Statistical models

## Abstract

Cognitive impairment is a primary feature of schizophrenia, with alterations in several cognitive domains appearing in the pre-morbid phase of the disorder. White matter microstructure is also affected in schizophrenia and considered to be related to cognition, but the relationship of the two is unclear. As interaction between cognition and white matter structure involves the interplay of several brain structures and cognitive abilities, investigative methods which can examine the interaction of multiple variables are preferred. A multiple-groups structural equation model (SEM) was used to assess the relationship between diffusion tension imaging data (fractional anisotropy of selected white matter tracts) and cognitive abilities of 196 subjects - 135 healthy subjects and 61 patients with schizophrenia. It was found that multiple-indicators, multiple-causes model best fitted the data analysed. Schizophrenia moderated the relation of white matter function on cognition with a large effect size. This paper extends previous work on modelling intelligence within a SEM framework by incorporating neurological elements into the model, and shows that white matter microstructure in patients with schizophrenia interacts with cognitive abilities.

## 1 Introduction

Schizophrenia is often accompanied by impairment in general intelligence and in several cognitive domains (Weickert et al. 2000; Wells et al. 2015; Tandon et al. 2009). These include impairment in episodic memory (Leavitt and Goldberg 2009), processing speed (Knowles et al. 2010), verbal fluency (Henry and Crawford 2005), attention (Fioravanti et al. 2005), executive function (Orellana and Slachevsky 2013) and working memory (Barch and Smith 2008; Reichenberg and Harvey 2007). These impairments robustly contrast them with healthy subjects (Tandon et al. 2009; Reichenberg and Harvey 2007; Keshavan et al. 2008).

Cognitive dysfunction appears in the pre-morbid phase of the disorder and progresses, but with a highly variable course (Weickert et al. 2000). In the pre-morbid phase, the dysfunction has an effect size of 0.5 (Woodberry et al. 2008), and predicts the severity of symptoms and functional outcome after onset (Wells et al. 2015), with impairments translating into poor social and occupational skills (Bowie et al. 2008).

Intelligence requires the proper integration of multiple brain areas (Jung and Haier 2007; Deary et al. 2010), one of the principle roles of white matter. The integrity of white matter is often measured using diffusion tensor imaging (DTI), and a large and growing body of DTI studies have reported white matter tract differences in patients with schizophrenia (Keshavan et al. 2008; Chua et al. 2007; Kanaan et al. 2009; Li et al. 2009; Wagner et al. 2015). Fractional Anisotropy (FA) is perhaps the commonest DTI measure employed in this regard, reflecting white matter micro- and macrostructural organisation, and myelination, and has consistently been shown to be reduced in schizophrenia. Mega-analyses (Kanaan et al. 2017, Kelly et al., 2017) and meta-analyses (Vitolo et al. 2017, Bora et al. 2011, Yang et al. 2017) alike confirm FA to be lower than in healthy controls, related to symptoms, and present from first onset (Samartzis et al. 2014). As in the healthy (Ohtani et al. 2017), FA has been shown to be related to various measures of cognition in patients with schizophrenia (Knochel et al., 2016, Alloza et al. 2016, Hidese et al. 2017).

This paper aims at extending these findings, now focusing on clarifying whether the relation between white matter structure and cognition differs in patients with schizophrenia as compared to health subjects. The presence of this difference might suggest white matter is involved in the cognitive change in schizophrenia. A multiple groups Structural Equation Modelling (SEM) approach was used, as it is an optimal method to evaluate the relationship between several variables, and to design and test a model representing these relations. The model was specified with one latent variable, which was correlated with white matter structural measurements and to neuropsychological test scores. Based on the aforementioned cognitive impairment in patients with schizophrenia, we hypothesised that schizophrenia would moderate the effect of white matter function on cognition and that this moderation would be reflected in the latent variable mean, i.e., we expected a large effect size when comparing the mean of the latent variable between the groups.

## 2 Methods

### 2.1 Subjects

The patients were recruited from the inpatient and outpatient clinics of the South London and Maudsley Hospital National Health Service (NHS) Trust. An experienced psychiatrist established the diagnosis of schizophrenia (DSM-IV criteria) using semi-structured interviews and detailed case-note review. The control subjects were matched to the patient group for age, gender, handedness and premorbid IQ using the National Adult Reading Test (Russell et al. 2000). Exclusion criteria for the control group were personal history of mental illness or family history of psychotic illness. Exclusion criteria for both groups were history of head injury with loss of consciousness, neurological illness or current drug or alcohol dependence. Sixty-one subjects with schizophrenia and 135 healthy subjects were assessed using Wechsler's Adult Intelligence Scale (WAIS-III) (Wechsler 1995) prior to MRI scanning (1.5 Tesla MRI). Fractional anisotropy of 48 white matter tracts was acquired. This dataset has previously been analysed for gender differences (Kanaan et al. 2012) and for differences on FA anisotropy between schizophrenics and healthy subjects (Kanaan et al. 2017). The control group was 57% male, the patient group 82% male; the mean years of education in the control group was 14.71 and 12.98 in the patient group (Table 1).

**Table 1:** Characteristics of the subjects.

Variables	Healthy subjects (mean, n = 135)	Patients (mean, n = 61)	p-Value
Age	28.742	33.207	0.012^a^
Gender			
Male (%)	57%	82%	<0.001^b^
Female (%)	43%	18%	
Age of onset	NA	20.82	
Education (mean, in years)	14.71	12.98	<0.001^a^
Handedness			
Right	127	60	
Left	6	0	0.212^b^
Mixed	1	1	
WAIS-III verbal	108.3 (sd 13.97)	100.6 (sd 14.63)	0.001^a^
WAIS-III performance	109.1 (sd 11.68)	97.3 (sd 17.27)	<0.001^a^

n, number of patients; (*) For one patient of the healthy group handedness was not recorded.

a Wilcoxon-Mann-Whitney test was used for the continuous variables.

b The correlation of the categorical variables was carried out using Fisher exact test.

### 2.2 Neuroimaging

A GE Signa 1.5 Tesla LX MRI system (General Electric, Milwaukee, Wisconsin, USA) was used, with a standard birdcage quadrature and an echo planar imaging sequence peripherally gated to the cardiac cycle. Sixty-four images with diffusion gradients (b = 1300 s/mm^2^) were acquired together with seven non-diffusion-weighted images (b = 0). The diffusion gradients were uniformly distributed in space (Jones et al. 2002) at each of 60 slices; TR was 15 cardiac R-R intervals with a TE of 107 ms. The acquisition gave isotropic (2.5 mm^3^) voxels, which were reconstructed to a 1.875 × 1.875 mm in-plane pixel size. Mutual-information image correction was applied, then non-brain tissue was removed and finally fractional anisotropy in each remaining voxel was calculated using in-house software (Catani et al. 2002).

Image processing was conducted using TBSS v1.2 (Smith et al. 2006). The FA images were all aligned to the Johns Hopkins University - International Consortium of Brain Mapping DTI-81 white matter atlas (JHU DTI atlas) (Mori et al. 2008) with FNIRT in FSL (http://fsl.fmrib.ox.ac.uk/fsl/fslwiki/). The “skeletons” of the FA images were thresholded for white matter (FA > 0.3) and projected onto the mean of all the FA skeletons. They were further subdivided according to 48 JHU DTI atlas regions, with FA averaged per region per-subject and these regional means compared between groups using IBM SPSS v20 (www.ibm.com/software/analytics/spss).

### 2.3 Statistical package and assumption testing

The analysis was carried out using R (version 3.3.2). The R lavaan package (version 0.5-23.1097) was used to specify the models and later run the structural equation analysis (Rosseel 2012). The MVN package (version 4.0.2) was used to test normality (Korkmaz, Goksuluk, and Zararsiz 2014).

We had measures for 48 tracts, but focussed on a subset of these in order to follow Kline, 2004a, Kline, 2004b recommendation of 10 to 20 observations per indicator (Kline 2004a). The decision on inclusion of variables used a data-driven approach, which consisted of fitting a multiple indicators, multiple causes (MIMIC) model similar in structure to those in Fig. 3, but including all available tract measures and cognitive variables, which were then ranked using a modification indices test. This test is part of the lavaan package and it iteratively removes one indicator at a time from the model further calculating the difference that this removal causes to the chi-square value. Each indicator is then ranked by how much it changes the chi-square. We used only tracts from the right hemisphere, the resulting set of tracts comprising the uncinate, external capsule, superior corona radiata, cingulum body, cingulum hippocampus, superior cerebellar pedunculum and the superior fronto-occipital fasciculum.

We followed standard notation in our diagrams. Boxes were used to represent indicators/variables, circles to represent latent variables and arrows to represent regressions. In the interest of clarity, residuals and correlations between the neurological tracts were not represented in the diagrams. A histogram with the distribution of the variables is available in Fig. 2.

### 2.4 Assumption testing

**Fig. 1:**
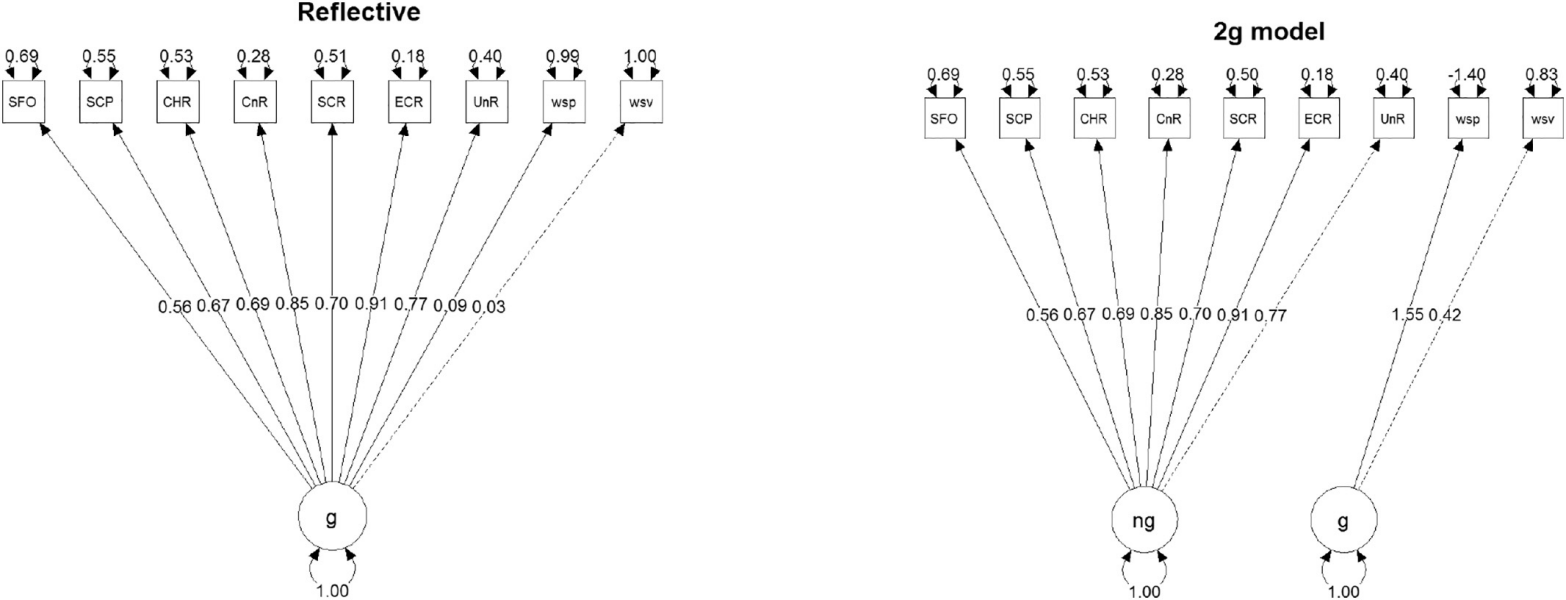
Diagrams for reflective and the 2 g model. See Table 2 for the corresponding fit indices. Each arrow is accompanied with its factor loadings or standardized coefficients. Residuals are not shown. The n-indicators: UnR, right uncinate; ECR, right external capsule; SCR, right superior corona radiata; CnR, right cingulum; CHR, right cingulum (hippocampus); SCP, right superior cerebellar pedunculum; SFO, right superior fronto-occipital fasciculus. P-indicators: wsp, WAIS-III performance; wsv, WAIS-III verbal.

A concern that besets SEM analysis is confirmation bias. One way of approaching this issue is by analysing semi-equivalent models and picking the one that fares best both in representing a theory about the relations, and by its performance in fit tests (Kline 2004b). We tested three equivalent models (Reflective, 2g, and MIMIC, see Fig. 1), which Kievit (2012) previously tested using psychological measures in healthy subjects. Psychological and neurological measures will be referred to as p- and n-indicators, respectively.

**Fig. 2:**
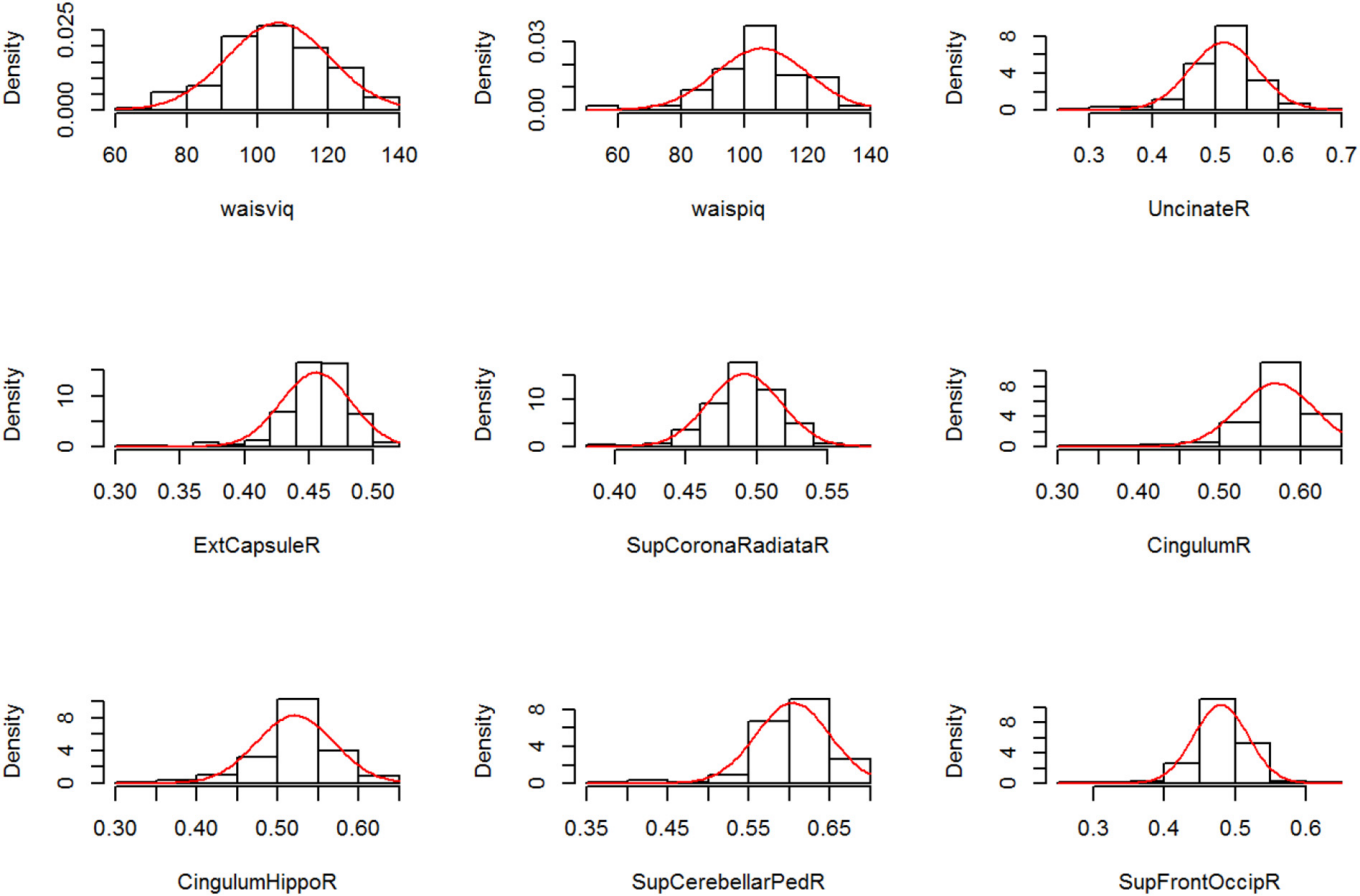
Histogram for all used variables. UncinateR, right uncinate; ExtCapsuleR, right external capsule; SupCoronaRadiataR, right superior corona radiata; CingulumR, right cingulum; CingulumHippoR, right cingulum (hippocampus); SupCerebellarPedR, right superior cerebellar pedunculum; SupFrontOccipR, right superior fronto-occipital fasciculus; waisviq, WAIS-III verbal; waispiq, WAIS-III performance.

**Fig. 3:**
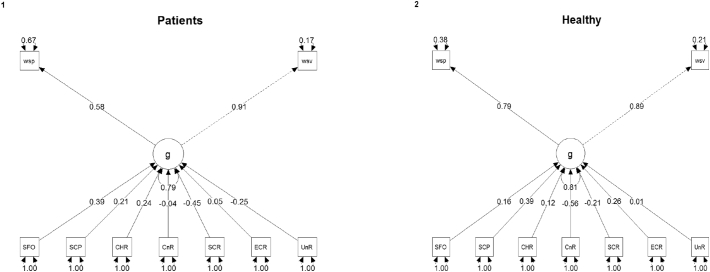
Diagrams for multiple groups SEM. Each arrow is accompanied with its factor loadings and standardized coefficients. Residuals are not shown. The n-indicators: UnR, right uncinate; ECR, right external capsule; SCR, right superior corona radiata; CnR, right cingulum; CHR, right cingulum (hippocampus); SCP, right superior cerebellar pedunculum; SFO, right superior fronto-occipital fasciculus. P-indicators: wsp, WAIS-III performance; wsv, WAIS-III verbal.

The first model was specified with a latent variable that reflects the variance of all indicators (Reflective model, Fig. 1). In the second model, two latent variables were specified, each either picking up variance from the n-indicators or from the p-indicators (2 g model, Fig. 1). The third is a MIMIC model, with n-indicators that form the latent variable, which in turn is reflected by the p-indicators (not shown, but with similar specification as the ones in Fig. 3).

The semi-equivalent models were fitted using the total dataset comprising patients and healthy subjects, and the three models converged to a solution with a standard maximum likelihood estimation. Chi-square is a measure of exact fit, and when it is not significant the model is consistent with the covariance data (Kline 2004b) and is said to fit. The reflective model (Fig. 1) yielded a chi-square (p-value) of 235.7 (p ≤ 0.001), the 2 g model a chi-square of 129 (p ≤ 0.001) and for the MIMIC model 5.609 (p = 0.468). All fit indices improved in the MIMIC model, as compared to the reflective or the 2 g. ANOVA tests were used to gauge the significance of the differences of fit between the models (Table 2). Since the MIMIC model presented with best fit, we proceeded to the multiple group analysis using this model.

**Table 2:** The fit indices for the three semi-equivalent models.

Model	df	AIC	BIC	Chisq	Chisq diff	Df diff	Pr (>Chisq)
MIMIC	6	−2679	−2643	5.609	NA	NA	NA
2g	26	−2539	−2477	129	123.4	20	<0.001
Reflective	27	−2435	−2376	235.7	106.7	1	<0.001

df, degrees of freedom; AIC, Akaike Information Criterion; BIC, Bayesian Information Criterion; Chisq, chi-square; Chisq diff, chi-square difference; DF diff, degrees of freedom difference; Pr(>Chisq), p-value of the chi-square difference test (ANOVA).

## 3 Results

### 3.1 The multiple group analysis

A single-group SEM can reveal the proportionality and strength of individual correlations in a matrix of correlations; a multiple group SEM further allows for a comparison of both the fit of the models and the individual paths between the groups. Finally, one can calculate the effect size of the latent variable mean changes between groups.

The multiple group analysis involves several steps, starting with fitting the model to the data. The multiple group MIMIC model converged to a solution with a chi-square of 8.206 (p = 0.769), meaning that the model presented good fit in terms of chi-square.

Before proceeding to the analysis of the multiple group MIMIC model, one last assumption should be tested. To show how reliably one can assert that the attribute being measured in each group is the same (cognition, represented by the latent ng variable in Fig. 3), we performed a test of invariance between the groups (Finch and French (2015). This is tested by progressively constraining the model's loadings, intercepts and means to equality: we found invariance to the level of metric invariance, when loadings were constrained (tested with ANOVA, Table 3).

**Table 3:** Invariance testing.

Model	df	AIC	BIC	Chisq	Pr (>Chisq)
Configural	12	−2750.6	−2665.4	8.2065	
Equal loadings	13	−2752.6	−2670.7	8.2066	0.0001 (0.99)
Equal loadings and intercepts	14	−2748.6	−2670.0	14.2058	5.9992 (0.01)⁎

The fit indices for the invariance testing. df, degrees of freedom; AIC, Akaike Information Criterion; BIC, Bayesian Information Criterion; Chi-square difference, difference between the chi-square of the model with the previous one; Pr(>Chisq), p-value of the chi-square difference test (ANOVA).

⁎ p < 0.05.

The specification of the multigroup analysis is shown in Fig. 3 and the coefficients/loadings are listed in Table 5. An inspection of the coefficients shows that each indicator caused only a small change in the latent variable. In the patient group both the superior corona radiata and the superior fronto-occipital fasciculum's correlations to *g* were statistically significant; in the healthy group the cingulum and the superior cerebellar peduncle's correlations to *g* were statistically significant. For each standard deviation (sd) change in the superior fronto-occipital fasciculum tract (patient group) a change of 0.391 sd in *g* would be expected (Table 5). The same relationship in the healthy group was weaker (0.16 sd change in *g* for each 1 sd change in the variable).

The factor loadings showed an asymmetry in the patient group. For each change of one sd in the latent variable *g*, 0.575 sd change would be expected in the WAIS-III performance score, whereas the load on the WAIS-III verbal score amounted to 0.910. In the control group, it was found that for each 1 sd change, 0.788 sd would be expected to change on the performance score of WAIS-III test and approximately the same loaded into the verbal score (0.886). R^2^ values were as follows: in the control group WAIS-III verbal = 0.785, WAIS-III performance = 0.622, *g* = 0.190; and for the patient's group WAIS-III verbal = 0.829, WAIS-III performance = 0.331, *g* = 0.215.

We proceeded with a comparison of the means of the latent variable *ng* between groups using the approach proposed by Finch and French (2015). The procedure consisted of fitting the MIMIC model twice: first with loading, intercept and latent variable variances and means constrained to equality, and then with the same constraints but the means (Table 4). The means are free to vary, thus allowing their comparison between groups. As expected, the model fit degraded with the strict constraints and the means' difference between the groups wasn't statistically significant. Similar to our previous approach (Castro-de-Araujo and Kanaan 2017) we calculated an effect size estimate similar to Cohen's d (Finch and French 2015; Hancock 2001): the formula consists of the difference of latent means between groups (37.272 – 0) divided by the square root of the variance of the latent (84.476), which in this multiple group analysis gave an effect size of 4.05.

**Table 4:** Latent variable means comparison.

Model	df	AIC	BIC	Chisq	Chisq diff	Df diff	Pr(>Chisq)
Means varying freely	17	−2725	−2656	44.28	NA	NA	NA
Loadings, intercepts, residuals, lv. variances, and means constrained	18	−2725	−2660	45.39	1.113	1	0.2914

The fit indices for the comparison between the all-constrained model and the same model with means let to vary freely. The difference between the two models is not statistically significant in a comparative anova. df, degrees of freedom; AIC, Akaike Information Criterion; BIC, Bayesian Information Criterion; Chisq diff, difference between the chi-square of the model with the previous one; DF diff, degrees of freedom difference; Pr(>Chisq), p-value of the chi-square difference test (ANOVA).

**Table 5:** Standardized coefficients for each indicator per group.

Tract	Patients				Healthy			
	std	est	se	p-Value	std	est	se	p-Value
Standardized coefficients								
Uncinate	−0.250	−62.085	48.152	0.197	0.012	2.951	31.874	0.926
External capsule	0.047	22.215	121.33	0.854	0.256	118.486	82.369	0.150
Superior corona radiata	−0.453	−230.276	97.312	0.017*	−0.211	−101.918	64.855	0.116
Cingulum	−0.041	−12.223	58.775	0.835	−0.564	−144.546	42.036	0.001*
Cingulum hippocampus	0.239	65.082	47.860	0.173	0.124	32.125	33.906	0.343
Superior cerebellar pedunculum	0.211	62.793	54.887	0.252	0.392	105.644	32.746	0.001*
Superior fronto-occipital fasciculus	0.391	122.023	55.543	0.028*	0.158	53.379	39.509	0.176

Standardized factor loadings for the latent variable ng								
WAIS-III verbal	0.910	1.000	0		0.886	1.000	0	
WAIS-III performance	0.575	0.746	0.307	0.015*	0.788	0.744	0.137	0.000*

Std, standardized coefficient; est, estimate; se, standard error.

There was no significant difference between latent variable means across groups, but we noted a trend towards stronger correlations in the healthy group, as four out of six correlations were stronger in this group, and we decided to test this more explicitly. We fit a last model in which all the n-indicators were constrained to equality between groups and compared it with the model without these constraints with ANOVA (See Table 6). As the difference was statistically significant, the paths were distinct between groups, showing that schizophrenia moderates these correlations.

**Table 6:** Multigroup models, comparison with and without structural model constrained to equality.

	Df	AIC	BIC	Chisq	Chisq diff	Df diff	Pr(>Chisq)
Multigroup SEM	12	−2751	−2665	8.206	NA	NA	NA
Multigroup constrained	25	−2744	−2702	40.37	32.16	13	0.002

Both models have similar specifications, but one has all the n-indicators (see text) constrained to equality between groups (healthy and patients), thus allowing us to state that the significant difference between models must be due to the structural model (n-indicators, pointing to *g*).

## 4 Discussion

We found that schizophrenia moderates the correlation between white matter tract integrity and cognition. It is possible to identify a trend towards stronger correlations between n-indicators and *g* in the healthy group of the MIMIC model, suggesting that schizophrenia moderates the effect of white matter on cognition by weakening the correlations between the n-indicators and the latent variable *g*. This was shown by means of a model comparison ANOVA test where one of the models had all the n-indicators constrained to equality across groups. The effect size of the moderation of schizophrenia on the mean of the latent variable *g* was large, but the difference was not significant.

The models we tested were designed to sensibly represent how physical alterations, such as tract connectivity, relate to cognitive functioning. Our results corroborate previous findings in both healthy subjects and in subjects with early psychosis and suggest that representing neuro-psychological data with MIMIC models is significantly superior to single level models (as the tested Reflective and 2 g) (Castro-de-Araujo and Kanaan 2017; Kievit 2011). This points towards a preferred model for neurocognitive data with latent variable statistics in the future (for a sensible discussion on this refer to Kievit 2011; Barrett 2011; Vul 2011; Berkman and Lieberman 2011; Bagozzi 2011).

We hypothesised that the differences in the means of the latent variable *g* would be significant (based on our recent finding from a dataset comprising subjects with early psychosis and grey matter volumes (Castro-de-Araujo and Kanaan 2017), however this was not the case. Overall, schizophrenia strongly interferes with this rather simple model of the brain/cognition relationship: it had a large effect (4.05) on the mean value of *g*. Furthermore, the difference in coefficients across groups was statistically significant. This means that schizophrenia produces changes in how white matter integrity correlates to cognition.

The clinical relevance of this finding is twofold. First, it would suggest that interventions should aim both at white matter integrity and at cognitive recovery of subjects with the disorder. Secondly, it is compatible with recent attempts to classify disorders as true dimensional constructs (Castro-de-Araujo and Kanaan 2017), by its incorporation of neuroimaging findings into a construct for cognition.

Methodologically, SEM can potentially offer a fine-grained picture of the relationships between brain changes and cognition. One can evaluate constructs that cannot be directly measured, as well as inspect and test the multiple specific correlations included in the model. We specified a latent variable that incorporates both cognitive and neurological information in this paper, but the technique can be applied to specify psychiatric conditions as latent. MIMIC models have again been shown to be superior to others in terms of representing neuropsychology data, confirming previous findings from our group. Latent variable statistics are important in psychiatry research, because they allow us to treat the disorders as continua.

The findings should be interpreted with caution however, due to the following limitations. Firstly, the groups were different in education and age, either of which may contribute to differences in FA. Secondly, the sample was not multivariate normal, and non-normality interferes with the multiple likelihood estimation, potentially inflating the chi-square (Benson and Fleishman 1994). Thirdly, the neuroanatomical atlas used in this study has recently faced concerns regarding the stability of its labels across versions (Rohlfing 2013). The use of latent variable statistics in psychiatry will face difficulties regarding how to decide which variables/indicators to include and which not to include in the models. This results from the fact that we often do not have access to large datasets (although this might improve in the future), and that neuroimaging techniques are able to offer measurements of numerous brain tracts or grey areas. Since it is necessary to keep the model within a ratio of 10–20 indicators per observation, some method for deciding what to include must be used. The method we used was a data-driven one (modification indices test from the lavaan package), not hypothesis driven. Hopefully, with larger data sets this will become less of a problem for future research. Finally, one should be cautious while generalising from these results, as the available sample was relatively small.

## Conflicts of interest

The authors have declared that there are no conflicts of interest in relation to the subject of this study.

## Contributors

Author Castro-de-araujo designed the study and run the analysis. Authors Mathew Allin, Marco Piccioni, Colm Mcdonald, and Christos Pantelis helped with the manuscript. Prof. Richard helped with the manuscript, guidance and English proof-reading. All authors contributed to and have approved the final manuscript.

## Role of the funding source

CAPES Foundation (Ministry of Education of Brazil) supported Castro-de-araujo during his PhD.
